# Adaptation and validation of the Chinese version of the New Sexual Satisfaction Scale–Short Form in a sample of Chinese women

**DOI:** 10.1093/sexmed/qfad065

**Published:** 2023-12-29

**Authors:** Chanchan Wu, Edmond Pui Hang Choi, Pui Hing Chau, Aleksandar Štulhofer

**Affiliations:** School of Nursing, Li Ka Shing Faculty of Medicine, The University of Hong Kong, Hong Kong, China; School of Nursing, Li Ka Shing Faculty of Medicine, The University of Hong Kong, Hong Kong, China; School of Nursing, Li Ka Shing Faculty of Medicine, The University of Hong Kong, Hong Kong, China; Department of Sociology, Faculty of Humanities and Social Sciences, University of Zagreb, Zagreb, 10000, Croatia

**Keywords:** sexual satisfaction, female sexual health, cross-cultural adaptation, validation

## Abstract

**Background:**

Existing research on sexual satisfaction has been carried out primarily in Western samples, lacking insights from less sexually permissive cultures such as China, particularly in the case of heterosexual and sexual minority women.

**Aim:**

This study aimed to culturally adapt and validate the New Sexual Satisfaction Scale–Short Form (NSSS-S) in a sample of Chinese women with diverse sexual identities.

**Methods:**

The standard forward-backward translation procedure and cognitive debriefing were conducted to translate the NSSS-S into Mandarin Chinese (NSSS-SC). The psychometric properties of the NSSS-SC were assessed in this cross-sectional survey.

**Outcomes:**

The primary outcome measure was the NSSS-S. The World Health Organization Quality of Life–Abbreviated Form, the Positive Sexuality Scale, the Female Sexual Function Index, and a single-item measure of sexual satisfaction were used to test the measure’s validity.

**Results:**

A total of 336 Chinese women were recruited, with an average age of 26.82 years (SD, 6.03; range, 18-56). The 1-dimensional model had a good fit to the data and was invariant across 2 sexual identity groups (cisgender heterosexual and sexual minority women) and age. The NSSS-SC had good reliability; no significant floor and ceiling effects were observed. We found moderate to strong correlations between the NSSS-SC scores and the sexual satisfaction, sexual function, positive sexuality, and quality-of-life indicators. On average, cisgender heterosexual women were characterized by higher sexual satisfaction scores when compared with sexual minority women.

**Clinical Translation:**

The NSSS-SC can be used as a reliable and culturally appropriate measure of sexual satisfaction in women of different ages and sexual identities, which could be used in future sex-related surveys.

**Strengths and Limitations:**

This study is the first to translate the NSSS-S into Chinese and validate the NSSS-SC in a sample of Chinese women with varied sexual identities. However, this study recruited only female participants; future studies should also validate the NSSS-SC in Chinese men.

**Conclusion:**

The NSSS-SC is linguistically equivalent to the original scale and has solid psychometric properties, which can be used to assess sexual satisfaction levels in diverse samples of Chinese women.

## Introduction

Sexual satisfaction (SS) is a key factor affecting individual sexual health as well as overall well-being.^1^ The most accepted definition of SS was proposed by Lawrance and Byers^2^ as “an affective response arising from one’s subjective evaluation of the positive and negative dimensions associated with one’s sexual relationship.” A systematic review revealed that physical and psychological health is associated with SS,^1^ with sexually satisfied women describing better psychological general well-being^3^ and with higher levels of SS being associated with better quality of life, higher self-esteem, and fewer mental and physical health problems.^3^^,^^4^ In addition, SS is closely related to overall relationship satisfaction.^5^

SS has been studied extensively over the past few decades; its importance has been emphasized by the World Health Organization’s definition of sexual health, which includes pleasure and satisfaction as essential elements.^6^ Over time, several instruments have been developed to measure SS,^7-9^ but most measures may be limited in their application by their focus on a specific status (being in a committed relationship) or multiple and distinct constructs (satisfaction, distress, or dysfunction). Furthermore, no scale other than the New Sexual Satisfaction Scale (NSSS)^10^ was designed to take the cross-cultural aspect and sexual minority populations into account. The NSSS has been translated and validated in various languages^11^ and recommended for use as “psychometrically valid and reliable option.”^9^

A review summarized that SS is a relevant construct in terms of positive sexuality and that different positive aspects of sexuality could improve mental and physical health as well as overall well-being.^12^ In addition, the link between SS and quality of life has been demonstrated cross-culturally, including Chinese women.^13^ Regarding the association between SS and female sexual function, many validation studies have examined the correlation by using the Female Sexual Function Index (FSFI) as a criterion to assess validity,^14^^,^^15^ while currently no similar assessment has been performed in the validation of the NSSS–Short Form (NSSS-S). Notably, the use of single-item measures in assessing homogeneous constructs is qualified,^16^ and the single-item SS measure has been recognized as an economic tool that meets some established psychometric criteria.^9^ Thus, these 3 measures, with the single-item SS measure, would be appropriate in assessing the validity of the NSSS-S.

Considering that some studies suggested that people in same-sex relationships may define “sex” somewhat differently than heterosexual individuals,^17^ the fact that the NSSS was originally validated for use in sexually diverse populations is an advantage.^10^^,^^18^ The NSSS-S, a short 1-dimensional version of the NSSS was subsequently developed as a more practical tool for use in a broader range of surveys.^19^ The NSSS-S has been translated and psychometrically tested in German,^20^ Portuguese,^21^ and Spanish.^22^ Overall, the NSSS-S validation studies have found that the measure is characterized with excellent reliability,^9^^,^^19^ while results regarding the validity were inconsistent.^9^^,^^19-22^ Although the NSSS-S has been translated into multiple languages, it lacks evidence of validity in China, a country with a conservative sexual culture. There are only a few Chinese studies on SS that used nonvalidated measures,^23-25^ so the current study aimed to translate, culturally adapt, and validate the Mandarin Chinese version of the NSSS-S (NSSS-SC).

## Methods

### Participants

This was a cross-sectional study conducted online in China. Eligible participants included Chinese adult women who (1) self-identified as women, (2) were able to read Mandarin Chinese, and (3) have had sex with ≥1 sex partners (with or without penetration). Participants who reported that they had never had sexual intercourse with other people were excluded from the analyses. The sample size was calculated according to the recommendations used for validation studies, with 10 to 20 respondents per item and an absolute minimum of 100 to 250, depending on the number of items.^26^ Given that the NSSS-S has 12 items, 240 participants were considered an adequate sample size.

A total of 431 sexually experienced participants were recruited, but only those who reported at least 1 sexual relationship were included in the analyses (*N* = 336). In the final sample, 157 (46.7%) were cisgender heterosexual women and 179 (53.3%) were sexual minority women.

### Procedure

#### Stage 1: Translation and cross-cultural adaptation

After obtaining permission from the original author,^10^^,^^19^ the translation and cultural adaptation of the NSSS-S were conducted according to the cross-cultural adaptation guidelines for self-report measures.^27^ This stage involved 6 steps: forward translation, synthesis of the initial translations, backward translation, expert reviews, proofreading and finalization, and pilot testing and cognitive debriefing (supplementary material 1). The final Chinese version (NSSS-SC; supplementary material 2) was then ready to be psychometrically tested.

#### Stage 2: Psychometric properties of the NSSS-SC

Convenience and snowball sampling methods were used to recruit participants, with various methods used to reach the desired sample size, including recruitment through several nongovernmental and social innovation organizations that target the female population or sexual minority women in mainland China, respondent-driven recruitment by encouraging existing participants to recruit their peers,^28^ and online recruitment by posting a study banner on several popular online communities. This study was approved by the institutional review board of the first author’s university. Electronic informed consent was obtained from all the study participants. Data were collected via an online survey platform (Wenjuanxing) between July and September 2021.

### Measures

#### Chinese version of the NSSS-S

As described before, the NSSS-S comprises 12 items, with a 5-point Likert-type scale ranging from 1 (not at all satisfied) to 5 (extremely satisfied). Considering that the original study found that the measure is unidimensional,^19^^,^^20^ the total NSSS-SC score was computed by summing all 12 items (range, 12-60), with higher scores indicating higher SS.^19^

#### Female Sexual Function Index

The FSFI is a 19-item self-report questionnaire measuring female sexual function,^29^ which contains 6 domains: desire, arousal, lubrication, orgasm, global satisfaction, and pain. The FSFI has been translated into Chinese and adapted to apply to all women regardless of sexual orientations,^30^ and this study used the adapted Chinese version of the FSFI.

#### Positive Sexuality Scale

The Positive Sexuality Scale (PSS) is a 1-dimensional 5-item scale designed to assess the level of positive sexual expression in adult women.^31^ The participant is asked to consider her actual sexual relationship and to rate how much each of the 5 aspects of sexual life is representative of her current sexual experience with the partner. We obtained authorization of the original author and the Chinese translator of the PSS to use the validated Chinese version.

#### World Health Organization Quality of Life–Abbreviated Form

The World Health Organization Quality of Life–Abbreviated Form (WHOQOL-BREF) is a generic 26-item quality-of-life measure comprising 4 domains: physical, psychological, social relationships, and environment quality.^32^ The psychometric properties of its Chinese version have been confirmed^32^ and used in this study.

#### Sociodemographic information

In addition to participants’ sexual identity, we collected their age, ethnicity, relationship status, education level, employment status, monthly income, sexual practices, and counseling experience. A single-item SS measure was also used in support of the convergent validity^9^: participants were asked to rate their overall satisfaction with sex life in the past 4 weeks using a 5-point scale that ranged from *very dissatisfied* to *very satisfied*.

### Statistical analysis

No missing information was observed in the data set. Descriptive statistics (percentage, means, and SD) were used to summarize participants’ demographic characteristics. Floor and ceiling percentages of a scale indicated percentages of participants with the lowest and highest full-scale scores. Floor or ceiling effects are considered present if the percentages are >15%.^33^

Confirmatory factor analysis (CFA) was used to evaluate the original structure of the NSSS-SC, and we fit the unidimensional model to the data.^19^ Because our data were ordinal and violated multivariate normality (ie, model fit was inadequate with the maximum likelihood estimator even though all factor loadings were in the 0.69-0.86 range), we used a diagonally weighted least squares (DWLS) estimator based on polychoric correlations.^34^ Multiple goodness-of-fit indices were used to assess model fit, including root mean square error of approximation (RMSEA), comparative fit index (CFI), and Tucker-Lewis index (TLI). Taking into account that fit indices such as CFI and RMSEA can have somewhat inflated values when DWLS is used, we also provide standardized root mean square residual (SRMR) values, which have been found to be unaffected by the choice of estimator.^35^ Referring to the widely cited evaluations of cutoff criteria, this study used the following guidelines for acceptable model fit^36^: RMSEA (90% CI) ≤0.06, CFI ≥0.95, TLI ≥0.95, and SRMR ≤0.08.

After identification of the most appropriate model by CFA, measurement invariance across sexual identity and age was assessed. Specifically, multiple-group CFA was used to test measurement invariance across the 2 sexual identity groups—namely, the heterosexual group and sexual minority group^37^—and the level of at least partial scalar invariance was required to directly compare NSSS-SC scores in different groups.^38^ Chi-square difference (Δχ^2^) was used to assess measurement invariance across sexual identity. To explore measurement invariance across age, we used a MIMIC model (multiple indicators, multiple causes) with an interaction approach that allowed for simultaneous testing of uniform and nonuniform differential item functioning.^39^^,^^40^ A chi-square likelihood ratio test was used to assess measurement invariance in age.

Reliability was estimated with McDonald’s omega coefficient^41^ and corrected item-total correlations.^42^ Reliability values ≥0.7 represent adequate internal consistency. A corrected item-scale correlation coefficient ≥0.4 was used as the threshold for adequate item correlations with the overall construct.

The measure’s validity was examined with Pearson’s correlations between the NSSS-SC and the FSFI, PSS, WHOQOL-BREF, and single-item SS measure, with coefficients >0.4 supporting adequate validity. The correlations were defined as strong (≥0.5), moderate (≥0.3 and <0.5), or weak (<0.3).^43^ Positive associations were expected between the NSSS-SC and all other measures.

Finally, we aimed to replicate the widely investigated differences among sexual identities by comparing the NSSS-SC latent mean (SE) scores of cisgender heterosexual women and sexual minority women. Statistical software packages SPSS version 27.0 (IBM Corp) and JASP version 0.18 (JASP Team) were used for data analysis.

## Results

Table 1 presents the sociodemographic and sexuality-related characteristics of the sample. Most women reported lifetime masturbation (82.7%) and penetrative sex (86.9%). Over half of surveyed women (67.6%) had a romantic partner. The majority of participants (94.3%) reported never seeking sexual health counseling.

**Table 1:** Participants’ sociodemographic and sexuality-related characteristics (*N* = 336).

**Variable: item/dimension**	**No. (%) or mean ± SD**
Sexual identities	
Cisgender heterosexual women	157 (46.7)
Sexual minority women	179 (53.3)
Cisgender homosexual women	54 (16.1)
Cisgender bisexual women	59 (17.6)
Cisgender pansexual women	37 (11.0)
Others (asexual/prefer not to be labeled, et al)	29 (8.6)
Age, y	
Group	
18-20	35 (10.4)
21-30	241 (71.7)
31-40	48 (14.3)
41-56	12 (3.6)
Overall sample (*N* = 336)	26.82 ± 6.03
Cisgender heterosexual women (*n* = 157)	28.37 ± 6.78
Sexual minority women (*n* = 179)	25.46 ± 4.91
At first masturbation	14.96 ± 6.00
At first sex	20.35 ± 3.51
Education	
High school/technical secondary school and below	14 (4.2)
College/bachelor	190 (56.5)
Graduate degree and above	132 (39.3)
Ethnicity	
Han	305 (90.8)
Other (Muslim, Zhuang, et al)	31 (9.2)
Employment	
Unemployed	28 (8.3)
Full-time student	111 (33.0)
Organization officer	31 (9.2)
Professional and technical personnel	76 (22.6)
Administrative personnel	15 (4.5)
Service personnel	29 (8.6)
Other (farmers, fishers, freelancers, et al)	46 (13.7)
Monthly income, CNY	
No income or <1000	77 (22.9)
1001–5000	80 (23.8)
5001–11 000	88 (26.2)
>11 000	91 (27.1)
Relationship status	
Single, including divorce	104 (31.0)
Multiple partners	5 (1.5)
Steady partner	227 (67.6)
Masturbation: lifetime	
No	58 (17.3)
Yes	278 (82.7)
Penetrative sex: lifetime	
No	44 (13.1)
Yes	292 (86.9)
Sexual counseling experience	
Yes	19 (5.7)
No	317 (94.3)
Sexual function: FSFI (range, 7.2–36.0)	
Desire	3.32 ± 1.06
Arousal	4.38 ± 1.02
Lubrication	5.07 ± 0.89
Orgasm	4.31 ± 1.07
Satisfaction	4.48 ± 1.16
Pain	4.94 ± 0.86
Total score	26.50 ± 3.97
Overall sexual satisfaction: single-item measure (range, 1–5)	3.53 ± 1.24
Positive sexuality: PSS (range, 5–35)	30.12 ± 6.09
Sexual satisfaction: NSSS-SC (range, 12–60)^a^	
Overall sample	40.94 ± 12.09
Cisgender heterosexual women	43.23 ± 10.96
Sexual minority women	38.94 ± 12.70

Abbreviations: CNY, Chinese Yuan; FSFI, Female Sexual Function Index; NSSS-SC, New Sexual Satisfaction Scale–Short Form (Chinese version); PSS, Positive Sexuality Scale.

a Composite (average) scores are shown.

### CFA of the NSSS-SC

The CFA findings of the NSSS-SC according to the DWLS estimator indicated that the original unidimensional model had a good fit to the data: χ^2^ = 57.60, *df* = 54, RMSEA = 0.014 (90% CI, 0.000-0.038), CFI = 0.999, TLI = 0.999, SRMR = 0.055 (Table 2). Factor loadings for all 12 items are presented in Table 3. All loadings were ≥0.69, indicating satisfactory structural validity. We repeated the analysis in a subsample of women who were sexually active in the past 6 months (*n* = 237), and the model fit was similar: χ^2^ = 60.80, *df* = 54, RMSEA = 0.023 (90% CI, 0.000-0.049), CFI = 0.998, SRMR = 0.070.

**Table 2:** Confirmatory factor analysis of the NSSS-SC and measurement invariance across sexual identity and age.

	**χ** ^**2**^ **(*df*)**	**CFI**	**RMSEA (90% CI)**	**SRMR**	**△χ** ^**2**^ **(△*df*)**	***P* value**
**Confirmatory factor analysis**						
Unidimensional model	57.60 (54)	0.999	0.014 (0.000–0.038)	0.055		
**Measurement invariance**						
Across sexual identity						
Configural	76.51 (108)	1.000	<0.001 (0.000–0.000)	0.059		
Metric	90.99 (119)	1.000	<0.001 (0.000–0.000)	0.067	14.48 (11)	.21
Scalar	99.34 (130)	1.000	<0.001 (0.000–0.000)	0.064	8.35 (11)	.68
Across age^a^						
Full model	2130.52 (240)					
Item 1	2135.07 (242)				4.55 (2)	>.05
Item 2	2134.74 (242)				4.22 (2)	>.05
Item 3	2133.50 (242)				2.98 (2)	>.05
Item 4	2132.95 (242)				2.43 (2)	>.05
Item 5	2135.88 (242)				5.36 (2)	>.05
Item 6	2135.32 (242)				4.80 (2)	>.05
Item 7	2186.32 (242)				55.80 (2)	<.001
Item 8	2135.16 (242)				4.64 (2)	>.05
Item 9	2136.14 (242)				5.62 (2)	>.05
Item 10	2134.76 (242)				4.24 (2)	>.05
Item 11	2132.98 (242)				2.46 (2)	>.05
Item 12	2134.45 (242)				3.93 (2)	>.05

Abbreviations: CFI, comparative fit index; NSSS-SC, New Sexual Satisfaction Scale–Short Form (Chinese version); RMSEA, root mean square error of approximation; SRMR, standardized root mean square residual.

a Each item is constrained.

**Table 3:** Reliability, factor loadings, and descriptive statistics for the NSSS-SC.

				**Responses, No. (%)**				
	**Corrected item-total correlation**	**Factor loading**	**Mean ± SD**	**Not at all satisfied**	**A little satisfied**	**Moderately satisfied**	**Very satisfied**	**Extremely satisfied**
Total scale^a^			40.94 ± 12.09					
1. The quality of my orgasms.	0.78	0.80	3.09 ± 1.23	54 (16.1)	33 (9.8)	126 (37.5)	76 (22.6)	47 (14.0)
2. My “letting go” and surrender to sexual pleasure during sex.	0.83	0.85	3.41 ± 1.25	43 (12.8)	22 (6.5)	96 (28.6)	104 (31.0)	71 (21.1)
3. The way I sexually react to my partner.	0.81	0.83	3.43 ± 1.24	38 (11.3)	27 (8.0)	97 (28.9)	100 (29.8)	74 (22.0)
4. My body’s sexual functioning.	0.76	0.78	3.52 ± 1.18	33 (9.8)	19 (5.7)	97 (28.9)	115 (34.2)	72 (21.4)
5. My mood after sexual activity.	0.82	0.84	3.56 ± 1.18	31 (9.2)	24 (7.1)	84 (25.0)	121 (36.0)	76 (22.6)
6. The pleasure I provide to my partner.	0.80	0.82	3.72 ± 1.16	27 (8.0)	17 (5.1)	74 (22.0)	123 (36.6)	95 (28.3)
7. The balance between what I give and receive in sex.	0.84	0.86	3.45 ± 1.22	34 (10.1)	34 (10.1)	85 (25.3)	112 (33.3)	71 (21.1)
8. My partner’s emotional opening up during sex.	0.81	0.83	3.61 ± 1.24	33 (9.8)	23 (6.8)	80 (23.8)	106 (31.5)	94 (28.0)
9. My partner’s ability to orgasm.	0.79	0.80	3.60 ± 1.25	36 (10.7)	21 (6.3)	77 (22.9)	111 (33.0)	91 (27.1)
10. My partner’s sexual creativity.	0.79	0.80	3.35 ± 1.22	40 (11.9)	26 (7.7)	113 (33.6)	90 (26.8)	67 (19.9)
11. The variety of my sexual activities.	0.77	0.78	3.20 ± 1.22	43 (12.8)	35 (10.4)	128 (38.1)	72 (21.4)	58 (17.3)
12. The frequency of my sexual activity.	0.68	0.69	3.01 ± 1.25	58 (17.3)	42 (12.5)	118 (35.1)	74 (22.0)	44 (13.1)

Abbreviation: NSSS-SC, New Sexual Satisfaction Scale–Short Form (Chinese version).

a McDonald’s omega = 0.96. Floor, *n* = 13 (3.9%); ceiling, *n* = 23 (6.8%).

### Measurement invariance across sexual identity and age

Configural, metric, and scalar invariance was assessed by multiple-group CFA across sexual identity (cisgender heterosexual group and sexual minority group), and the standard △χ^2^ tests indicated scalar invariance of the NSSS-SC across the 2 groups (Table 2). When latent mean scores were compared between the sexual identity groups, cisgender heterosexual women reported significantly higher SS than sexual minority women (0.35, SE = 0.11, *P* < .01).

Finally, according to the MIMIC model with interaction analysis,^39^^,^^40^ only 1 of the 12 items (item 7: “The balance between what I give and receive in sex”) exhibited substantial nonuniform differential item functioning (β = 0.72, *P* < .001), which indicated that the NSSS-SC was reasonably age invariant in this sample.

### Reliability

The NSSS-SC showed good internal consistency, with a McDonald’s omega value of 0.96. The corrected item-total correlation ranged from 0.68 to 0.84. No significant floor and ceiling effects were found for the NSSS-SC (floor = 3.9%, ceiling = 6.8%), as shown in Table 3.

### Validity

Correlations between the NSSS-SC on one hand and the single-item SS measure, PSS, FSFI (6 domains and total scale), and WHOQOL-BREF (4 domains and total scale) on the other are presented in Table 4. All associations are statistically significant and in the 0.18-0.59 range. The NSSS-SC was positively and strongly correlated with PSS (*r* = 0.54, *P* < .001) and FSFI (*r* = 0.57, *P* < .001) and moderately correlated with WHOQOL-BREF (*r* = 0.45, *P* < .001). Expectedly, the NSSS-SC was positively and strongly related to the SS subscale of the FSFI (*r* = 0.52, *P* < .001), and the coefficient was the largest among the associations between the NSSS-SC and the 6 FSFI domains.

**Table 4:** Correlations between NSSS-SC and 3 conceptually related measures.

**Scale: domains**	***r* value** ^**a**^
Single item: sexual satisfaction	0.54
PSS: positive sexuality	0.54
FSFI	
Desire	0.28
Arousal	0.48
Lubrication	0.34
Orgasm	0.37
Satisfaction	0.52
Pain	0.21
Total score	0.57
WHOQOL-BREF	
Physical	0.31
Psychological	0.37
Social relationship	0.51
Environment	0.34
Total score	0.45

Abbreviations: FSFI, Female Sexual Function Index; NSSS-SC, New Sexual Satisfaction Scale–Short Form (Chinese version); PSS, Positive Sexuality Scale; WHOQOL-BREF, World Health Organization Quality of Life–Abbreviated Form.

a Each correlation: *P* < .001.

## Discussion

The current study translated and culturally adapted the NSSS-S into Mandarin Chinese (NSSS-SC) and explored its psychometric properties in Chinese women to contribute to the understanding of SS among women from less sexually permissive cultures, especially sexual minority women. During the process of translation and acculturation, several discrepancies were found in the specific expression of sexual pleasure. Considering a lack of uniform semantically consistent translations of such terms in the Chinese context, culturally adjusted expressions have been made to adapt the scale items to China’s relatively conservative culture. The results demonstrated satisfactory reliability and validity of the NSSS-SC as a unidimensional measure and confirmed its invariance across age and sexual identity.

Our analysis supported the original single-dimensional structure of the NSSS-SC, in line with international research.^19^^,^^20^^,^^22^ The internal consistency of the measure was high. We observed no substantial floor or ceiling effects, suggesting that the NSSS-SC accurately measured SS without reducing possible variation.^44^^,^^45^ In addition, there was a strong and significant positive correlation between the NSSS-SC and the single-item SS measure, suggesting that construct validity was robust.

The validity of the NSSS-SC was indicated by the measure’s moderate to strong positive correlations with positive sexuality, quality of life, and sexual functioning. In regard to concurrent validity, our analysis followed the use of the satisfaction domain of the FSFI as the criterion in various cultural settings.^5^^,^^14^^,^^15^ The observed substantial relationship between the NSSS-SC and sexual functioning is consistent with previous studies,^46^ as is the positive correlation between SS and women’s quality of life.^18^^,^^47^ We observed a strong link between the NSSS-SC and the social relationship domain of the quality-of-life measure, which is compatible with review findings that women’s SS was associated with more stable relationships in 29 countries.^47^

The NSSS-SC was invariant across age and sexual identity in the current study, which enabled direct comparisons. We found that the average SS was different between heterosexual women and sexual minority women, with the former reporting substantially higher SS levels. This is in contrast to the Spanish study, in which homosexual women reported significantly higher levels of SS than their straight peers.^22^ It should be noted that in China, unlike Spain, same-sex marriage is not legal. It was not until 2001 that homosexuality was removed from the list of mental diseases in China.^48^ Several other studies, carried out in more sexually permissive countries than China, did not observe substantial differences in SS between heterosexual and sexual minority women.^49^^,^^50^

The current study has certain clinical implications. First, SS should be measured with a reliable and culturally appropriate measure, and to the best of our knowledge, the NSSS-SC is currently the only validated scale to be used in a range of sexuality- and sexual health–related surveys. Second, the NSSS-SC can be used in women of different ages and sexual identities, which is of substantial relevance to sexual health experts. Third, Chinese health providers need to be sensitized to the fact that SS is an integral part of health and personal well-being, although the positive aspects of sexuality are rarely considered among existing sex research in China.^51^

Several study limitations should be noted. First, due to the sampling strategy used in this study, participants were relatively young and highly educated, so the findings cannot be generalized to the Chinese female population. Second, our study design did not allow for exploring the test-retest reliability of the NSSS-SC, so this needs to be accomplished in future research. In addition, future studies should validate the NSSS-SC in Chinese men.

## Conclusion

The current study presented the translation and cultural adaptation of the Mandarin Chinese version of the NSSS-S. Our findings indicate that the NSSS-SC is suitable for exploring SS among Chinese women of different ages and sexual identities. The validity of the NSSS-SC for male and older participants, as well as its utility in clinical settings, remains to be explored in future studies.

## Supplementary Material

Supplementary_file_qfad065

## Data Availability

The authors confirm that the data supporting the findings of this study are available within the article and its supplementary materials.
