# Supplementary material for: Adaptation and validation of the Chinese version of the New Sexual Satisfaction Scale–Short Form in a sample of Chinese women
Source: Sex Med. 2023 Dec 29;11(6):qfad065. doi: 10.1093/sexmed/qfad065 (PMC10756855; doi:10.1093/sexmed/qfad065)
Supplement: Supplementary_file_qfad065 [file supplementary_file_qfad065.docx]

# Appendix 1 Translation and Cross-cultural Adaptation process of NSSS-SC (Chinese version of New Sexual Satisfaction Scale-Short form)

After obtaining the permission from the original author,^1,2^ the translation and cultural adaptation of the NSSS-S were conducted based on the Cross-Cultural Adaptation Guidelines for Self-report Measures,^3^ involving six steps:

1. Forward translation: two bilingual translators who were familiar with the instrument independently forward translated the questionnaire into Mandarin Chinese (CN), one is the first author, a qualified Sexual Health Counselor (provided CN translation A) and the other is an associate professor of nursing who specialized in scale translation (provided CN translation B);
2. Synthesis of the initial translations: another independent translator who is a professional bilingual translator reconciled the translation A and B, and the first consensus Chinese version of NSSS-S was obtained (CN version 1);
3. Backward translation: the translation from the CN version 1 to English (EN) was performed independently by two other bilingual translators with no knowledge of the original version of NSSS-S but with experience in cross-cultural scale adaptations, one is an assistant professor of nursing (provided the CN-EN version A) and the other is a PhD candidate in nursing (provided the CN-EN version B);
4. Expert reviews: the study committee consisted of the five translators and a senior researcher (the last author who is an assistant professor of nursing with extensive experience in sexual health) and all are proﬁcient in English and Chinese, and any differences among the CN-EN version A and B and the original NSSS-S were discussed to confirm the accuracy of the back translation. On this basis, the expert committee assessed the consistency of expressions and made modifications based on CN version 1, then established a pre-final version (CN version 2);
5. Proofreading and finalization: the pre-final version was proofread by 4 female participants not included in the data analysis to confirm the readability and cultural appropriateness of all items, then the study committee discussed until agreed on the semantic, idiomatic, and conceptual equivalence between the original and the final version;
6. Pilot testing and cognitive debriefing: 16 female participants who were not included in the final analysis were interviewed in the preliminary survey to test whether the instruction is clear and to clarify if there were any words in the final items that were not easily understood or deemed unacceptable. None of the 16 women had any difﬁculty understanding and completing the final Chinese version, making the NSSS-SC ready to be psychometrically tested.

In the forward translation process, discrepancies were identified between CN translation A and B in translating “sexual functioning” (item 4) into “性功能” or “性能力”, with the former focusing on physiological functions and the latter emphasizing individual ability and colloquialism. Following a discussion by the study committee, the phrase was translated as “性功能” which is consistent with the original meaning. In the backward translation, there is also inconsistency in item 3: “The way I sexually react to my partner”, with CN-EN version A translated as “The sexual mode I react to my partner” and CN-EN version B translated as “My response to sex partner”. After a discussion among four participants in the proofreading stage, additional clarifications were made to adapt to the relatively restricted Chinese culture of sexual expression (the final translation was: “我回应我伴侣的性表达方式”).

Regarding other cultural adaptations, several discrepancies were found in expressions of sexual pleasure, such as “letting go” (item 2), “surrender” (item 2), and “emotional opening up” (item 8). Although there are similar metaphors or figurative descriptions in Chinese, they are either too vague or not universal enough, making a completely semantically consistent translation impossible. Thus, following suggestions from participants included in the pilot, culturally appropriate expressions, such as “释放自我”, were used to accommodate for China’s relatively conservative sexual culture. After a further review of this pre-final version, the Chinese NSSS-S (NSSS-SC) was administered to the 16 subjects and finalized.

**References**

1. Brouillard P, Štulhofer A, Buško V. The New Sexual Satisfaction Scale and Its Short Form. In: Milhausen R, ed. *Handbook of Sexuality-Related Measures.* Fourth ed.: Routledge; 2020:495-497.

2. Stulhofer A, Busko V, Brouillard P. Development and bicultural validation of the new sexual satisfaction scale. *Journal of sex research.* 2010;47(4):257-268.

3. Beaton DE, Bombardier C, Guillemin F, Ferraz MB. Guidelines for the process of cross-cultural adaptation of self-report measures. *Spine (Phila Pa 1976).* 2000;25(24):3186-3191.

# Appendix 2 Chinese version of the NSSS-S (NSSS-SC)

**新版性满意度量表-中文简版**

想想您最近6个月的性生活，请对下面描述各项的满意度进行评定：

|  | **一点也不满意** | **有一点满意** | **一般满意** | **非常满意** | **极其满意** |
| --- | --- | --- | --- | --- | --- |
| 1. 我性高潮的质量。 | 1 | 2 | 3 | 4 | 5 |
| 1. 性行为中我能“释放自我”并享受性愉悦。 | 1 | 2 | 3 | 4 | 5 |
| 1. 我回应我伴侣的性方式。 | 1 | 2 | 3 | 4 | 5 |
| 1. 我身体的性功能。 | 1 | 2 | 3 | 4 | 5 |
| 1. 性行为后我的心情 | 1 | 2 | 3 | 4 | 5 |
| 1. 我提供给我伴侣的性愉悦。 | 1 | 2 | 3 | 4 | 5 |
| 1. 性行为中我付出的与我得到的之间的平衡。 | 1 | 2 | 3 | 4 | 5 |
| 1. 性行为中我伴侣情绪放得开。 | 1 | 2 | 3 | 4 | 5 |
| 1. 我伴侣达到性高潮的能力。 | 1 | 2 | 3 | 4 | 5 |
| 1. 我伴侣性方面的新奇性。 | 1 | 2 | 3 | 4 | 5 |
| 1. 我性行为的多样性。 | 1 | 2 | 3 | 4 | 5 |
| 1. 我性行为的频率。 | 1 | 2 | 3 | 4 | 5 |
